# Mining the *Arabidopsis thaliana *genome for highly-divergent seven transmembrane receptors

**DOI:** 10.1186/gb-2006-7-10-r96

**Published:** 2006-10-25

**Authors:** Etsuko N Moriyama, Pooja K Strope, Stephen O Opiyo, Zhongying Chen, Alan M Jones

**Affiliations:** 1 School of Biological Sciences and Plant Science Initiative, University of Nebraska-Lincoln, Lincoln, NE 68588-0660, USA; 2 Department of Agronomy and Horticulture, University of Nebraska-Lincoln, Lincoln, NE 68583-0915, USA; 3 Departments of Biology and Pharmacology, University of North Carolina at Chapel Hill, Chapel Hill, NC 27599, USA

## Abstract

To identify divergent seven-transmembrane receptor (7TMR) candidates from the *Arabidopsis thaliana *genome, multiple protein classification methods were combined, including both alignment-based and alignment-free classifiers. This resolved problems in optimally training individual classifiers using limited and divergent samples, and increased stringency for candidate proteins. We identified 394 proteins as 7TMR candidates and highlighted 54 with corresponding expression patterns for further investigation.

## Abstract 2

A combination of multiple protein classification methods is described and used to identify a minimum set of 54 candidate seven transmembrane receptors in *Arabidopsis thaliana*.

## Background

Seven-transmembrane (7TM)-region containing proteins constitute the largest receptor superfamily in vertebrates and other metazoans. These cell-surface receptors are activated by a diverse array of ligands, and are involved in various signaling processes, such as cell proliferation, neurotransmission, metabolism, smell, taste, and vision. They are the central players in eukaryotic signal transduction. They are commonly referred to as G protein-coupled receptors (GPCRs) because most transduce extracellular signals into cellular physiological responses through the activation of heterotrimeric guanine nucleotide binding proteins (G proteins) [1]. However, an increasing number of alternative 'G protein-independent' signaling mechanisms have been associated with groups of these 7TM proteins [2-5]. Thus, for precision and clarity, we refer to these proteins simply as 7TM receptors (7TMRs), and candidate proteins in organisms greatly divergent to humans are designated here as 7TM putative receptors (7TMpRs).

The human genome encodes approximately 800 or more 7TMRs, both with and without known cognate ligands (the latter are so-called orphan GPCRs); they thus constitute >1% of the gene complement [6,7]. More than 1,000 genes or 5% of the *Caenorhabditis elegans *genome are predicted to encode 7TMRs; the majority of them appear to be chemoreceptors [8]. Approximately 300 7TMR-encoding genes (about 1% to 2% of the genome) have been recognized in the *Drosophila melanogaster *genome [6,7]. Compared to such large numbers of 7TMRs found in animal genomes, very few 7TMpRs have been reported in plants and fungi. Only 22 *Arabidopsis *7TMpRs have been described so far. Fifteen of them constitute the 'mildew resistance locus O' (MLO) family, whose direct interaction with the G-protein α subunit (Gα) has not been shown [9,10]. While another 7TMpR, GCR1 [11], directly interacts with the plant Gα subunit GPA1 [12], it has been shown that GCR1 can act independently of the heterotrimeric G-protein complex as well [2]. Hsieh and Goodman [13] recently reported five expressed proteins predicted to have 7TM regions (heptahelical transmembrane proteins (HHPs) 1 to 5) but these, like the other 16, do not have candidate ligands. Finally, an unusual Regulator of G Signaling (RGS) protein (designated AtRGS1) has been predicted to have 7TM regions [14]. RGS proteins function as a GTPase activating protein (GAP) to de-sensitize signaling by de-activating the Gα subunits of the heterotrimeric complex. Because *Arabidopsis *seedlings lacking AtRGS1 have reduced sensitivity to D-glucose [2,14,15], the possibility exists that AtRGS1 is a novel D-glucose receptor having an agonist-regulated GAP function. Although we designate them 7TMpRs here, it should be noted that neither a ligand nor a full signaling cascade has been demonstrated yet for any of these plant proteins, and only for a barley MLO protein has the 7TM topology been experimentally confirmed [9].

None of the reported *Arabidopsis *7TMpR proteins share substantial sequence similarity with known metazoan GPCRs constituting six different subfamilies. It appears that plant 7TMpRs dramatically diverged from known metazoan GPCRs over the 1.6 billion years since the plant and metazoan lineages bifurcated. It should be noted that *Arabidopsis *GCR1 shares weak but significant similarity with the cyclic AMP receptor, CAR1, found in the slime mold [2,11,16]. There is also very weak similarity to the Class B Secretin family GPCRs. However, other than GCR1, currently used search methods have not robustly identified plant 7TMpR proteins as candidate GPCRs. This great sequence divergence highlights the need for new approaches to identify divergent 7TMR candidates in non-metazoan genomes.

The human genome contains 16 Gα, 5 Gβ, and 12 Gγ genes. In stark contrast, both fungi and plants have much simpler G-protein coupled signaling systems. For example, the *Arabidopsis *genome contains one canonical Gα, one Gβ, and two Gγ genes [17]. Similarly, a small number of G-proteins are found in fungi; there are two Gα, one Gβ, and one Gγ in *Saccharomyces cerevisiae *[18-20] while *Neurospora crassa *and some fungi have more genes encoding each subunit [21-23]. Therefore, it may be reasonable to assume that plants and fungi have fewer GPCRs than human, and while approximately 200 *Arabidopsis *proteins were predicted to have 7TM regions, sequence divergence precludes unequivocal assignment of any as an orphan GPCR [24,25]. However, at least 61 7TMpRs have been recently predicted from the plant pathogenic fungus *Magnaporthe grisea *genome [26], raising the possibility that more divergent groups of 7TMpR proteins likely remain undiscovered in non-metazoan taxa.

In this report, we describe our comprehensive computational strategy for identifying 7TMpR candidates from the entire protein sequence set predicted from the *A. thaliana *genome, and compile their tissue-specific expression and co-expression patterns with G-proteins. To take advantage of different approaches, we combined multiple protein classification methods, including more specific (conservative) alignment-based classifiers and more sensitive alignment-free classifiers, to predict candidate 7TMpRs in divergent genomes more effectively.

## Results and discussion

### Identifying 7TMpR candidates using various protein classification methods

Among many protein classification methods commonly used, the current state-of-the-art and most used is the profile hidden Markov models (profile HMMs) [27]. It is used to construct protein family databases such as Pfam [28,29], SMART [30,31], and Superfamily [32]. However, profile HMMs and other currently used classification methods such as PROSITE [33,34] and PRINTS [35,36] share an important weakness. These methods rely on multiple alignments for generating their models (patterns, profile HMMs, and so on). Generating robust multiple alignments is difficult or impossible when extremely diverged sequences are included in the analysis; 7TMRs are one such protein family whose sequence similarities between subgroups can be lower than 25%. Furthermore, alignments are generated only from known related proteins (positive samples), and, therefore, no information from negative samples (unrelated protein sequences) is directly incorporated in the model building process. Identifiable 'hits' are, therefore, constrained by initial sampling bias, which becomes reinforced when models are iteratively rebuilt from accumulated sequences. Consequently, the predictive power, especially the sensitivity, of these classifiers decreases when they are applied against extremely diverged protein families.

To overcome this disadvantage and to increase sensitivities against such non-alignable similarities, several 'alignment-free' methods have been proposed recently. These methods quantify various properties of amino acid sequences and convert them into a descriptor array. Once multiple sequences with different lengths are transformed into a uniform matrix, various multivariate analysis methods can be applied. Kim *et al*. [37] and Moriyama and Kim [38] used parametric and non-parametric discriminant function analysis methods. Karchin *et al*. [39] incorporated profile HMMs with support vector machines (SVMs) using the Fisher kernel (SVM-Fisher) so that negative sample information can be taken into account when training the classifier. SVMs can be applied with completely 'alignment-free' sequence descriptors, for example, amino acid and dipeptide compositions. Such alignment-free classifiers are shown to outperform profile HMMs as well as Karchin *et al*.'s SVM-Fisher [40,41] (PK Strope and EN Moriyama, submitted). Another multivariate method, partial least squares (PLS) regression, was used by Lapinsh *et al*. [42] with physico-chemical properties of amino acids. We recently re-evaluated the descriptors used with PLS and optimized them to discriminate 7TMRs from other proteins [43].

We applied these methods against the entire predicted protein sequence set derived from the *A. thaliana *genome. As shown in Table 1, among the 28,952 protein sequences, the Sequence Alignment and Modeling system (SAM), a profile HMM method, predicted only 16 (excluding one alternatively spliced gene sequence) as 7TMpR candidates. Fifteen of them are identified as MLO or similar to MLO and one as GCR1 in The Arabidopsis Information Resource (TAIR) [44,45]. It clearly shows that SAM is highly specific (discriminating) with no false positive, assuming that current annotations are correct. SAM failed to identify only one known MLO (MLO4: At1g11000). This protein, as well as AtRGS1 and five recently predicted 7TM proteins (HHP1-5), were among the 16 previously predicted *Arabidopsis *7TMpRs not included in the randomly sampled 500 7TMR training sequences (see Materials and methods). Thus, we concluded that the predictive power of SAM alone is insufficient to identify highly diverged and potentially novel 7TMpR sequences.

The results obtained by SAM were compared with those obtained by alignment-free methods. As shown in Table 1, alignment-free methods (LDA, QDA, LOG, KNN, SVM with amino acid composition (SVM-AA), SVM with dipeptide composition (SVM-di), and PLS with amino acid properties (PLS-ACC)) predicted 2,000 to 3,400 proteins as 7TMpR candidates, which is about 10% of the entire predicted *Arabidopsis *proteome and about 30% to 50% of all possible transmembrane proteins (6,475 proteins) [24,25]. These alignment-free methods clearly call many false positives, and need further optimization to improve their discrimination power.

One advantage of alignment-free methods to be noted is their sensitivity against short or partial sequences [37,38]. Many of the 28,952 protein sequences used in this study are based only on *ab initio *gene prediction results, and hence are likely to contain various types of errors. If only a part of a 7TMR protein is predicted correctly, alignment-free methods could have a better chance to identify it.

**Figure 1:**
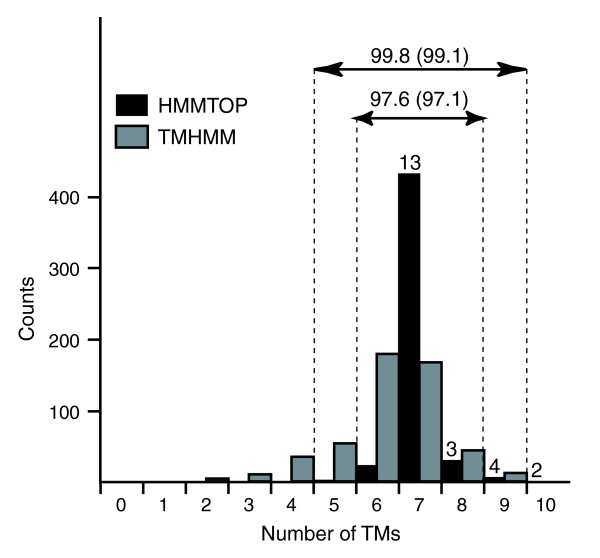
Distribution of transmembrane numbers predicted by HMMTOP (black bars) and TMHMM (gray bars) from the 500 7TMR sample sequences. Proportions (%) of the proteins predicted to have six to eight and five to nine TM regions by HMMTOP are shown at the top. The percentages shown in parentheses were obtained from the entire 7,674 7TMR dataset in GPCRDB. The numbers shown on the top of black bars are the number of previously predicted 22 *Arabidopsis *7TMpR proteins.

Table 1 lists *Arabidopsis *proteins that were predicted to have five to ten transmembrane regions and bins them by the number of transmembrane regions. HMMTOP 2.0 [46,47] predicted 201 proteins as having 7TM regions. This number is close to a previous prediction (184 proteins) [24,25]. We should note, however, that no single method predicts 7TM regions from all known 7TMRs exactly (see Materials and methods). As mentioned above, it is also possible that some deduced *Arabidopsis *proteins we analyzed do not contain the entire correct coding region. There were 952 *Arabidopsis *proteins predicted to have five to nine TM regions. Based on the distribution of predicted TM numbers obtained from the entire GPCRDB entries, this range (5 to 9 TM regions) could cover almost all of the 7TMR candidates (99.1%; see Figure 1 and Materials and methods). The 22 previously predicted *Arabidopsis *7TMpRs were predicted to have seven to ten TM regions (Figure 1). If we extend the range to 5 to 10 TM regions, the number of *Arabidopsis *7TMpR candidates becomes 1,179 proteins.

### Choosing 7TMpR candidates by combining prediction results

Among the ten alignment-free classifiers, LOG misclassified seven previously predicted *Arabidopsis *7TMpRs. KNN with *K *set at 5, 10, and 15 missed one, while KNN with *K *set at 20 classified them all correctly (see Materials and methods on KNN). To reduce the number of false positives (non-7TMRs predicted as 7TMRs) as well as false negatives (7TMRs predicted as non-7TMRs) and to obtain a set of 7TMpR candidates with higher confidence, we examined combinations of the prediction results by the remaining six alignment-free methods (LDA, QDA, KNN with *K *= 20, SVM-AA, SVM-di, and PLS-ACC). There were 652 proteins predicted as 7TMpR candidates by all six methods (by choosing the strict intersection). Using the number of predicted TM regions to be 5 to 10, 394 (342 after removing duplicated entries due to alternative splicing) proteins were identified as 7TMR candidates. These *Arabidopsis *proteins are listed in Additional data file 1. Of the 22 previously predicted 7TMpRs, 20 were found in this list. Although HHP4 and HHP5 were not included in this list, both were identified by two of the alignment-free methods: KNN and SVM-AA. Note that RGS1 and five HHP (as well as nine MLO and GCR1) sequences were excluded from the training set, and these six were not identified as candidate 7TMpRs by SAM.

A further restriction to protein topology of exactly 7TM regions and an amino-terminus located extracellularly reduced the candidate number to 64 (54 excluding duplications due to alternative splicing). This set included nine of the 22 previously predicted 7TMpRs. These 54 7TMpR candidates are the first targets for our further analysis and are summarized in Table 2 (also listed in Additional data file 2). Eighteen are described as simply 'expressed proteins' in the TAIR database (except for AT3G26090, which encodes RGS1). Interestingly, one of them (AT5G27210) is known to have weak similarity to a mouse orphan 7TMR. While others are known to belong to certain protein families (for example, MtN3 family), in many cases, their molecular functions have not been identified, and further investigation on these 7TMpR candidates is warranted.

The 54 proteins were grouped into families based on similarities to known protein sequences. Eight of the 54 7TMpR candidates, including GCR1 and RGS1, are encoded by single copy genes. In addition to the seven MLO proteins identified, there are eight MtN3 family members, two proteins of an unnamed family consisting of six expressed proteins, as well as multiple (two to three) members from smaller gene families (five or less). All members of the TOM3 family and the Perl1-like family, as well as the majority of the GNS/SUR4 family and an unnamed family consisting of five expressed proteins (expressed protein family 2) were included in the list. The identification of multiple members from these gene families using our alignment-free methods supported the consistency of this approach. However, for most of these families, not all members were found. Additionally, eight single representatives of small protein families consisting of two to five members and four single representatives of large protein families were found in the list. Some of these proteins, especially those from large protein families, may represent false positives as 7TMpR candidates. This 7TMR mining method can be refined, for example, by re-training models as well as using more flexible hierarchical classification.

The five predicted heptahelical proteins (HHP1-5) reported by Hsieh and Goodman [13] were identified by sequence similarity to human adiponectin receptors (AdipoRs) and membrane progestin receptors (mPRs) that share little sequence similarity to known GPCRs. HHP1-3 were identified in our initial list of 394 but were culled from the final list of 54 *Arabidopsis *7TMpR candidates. This is because HMMTOP predicted HHP1, HHP2, HHP4, and HHP5 to have seven TM regions and intracellular amino termini, in contrast to known GPCRs. This unusual structural topology was also found in AdipoRs [13,48]. HHP3 had eight predicted TM regions. Of the 15 MLO proteins, 8 were also predicted to have 8 to 10 TM regions by HMMTOP (Figure 1). Recently, Benton *et al*. [49] experimentally showed that *Drosophila *odorant receptors, another extremely diverged 7TMR family, have intracellular amino termini. Among our 394 candidate list, 23 proteins were predicted to have seven TM regions and intracellular amino termini (Additional data file 1). Therefore, we consider these 54 as a minimum working set of 7TMpR candidates, and many of the other proteins included in the list of 394 should be examined in the second stage.

### Expression patterns of genes encoding the 7TMpR candidates and G-protein subunits

**Figure 2:**
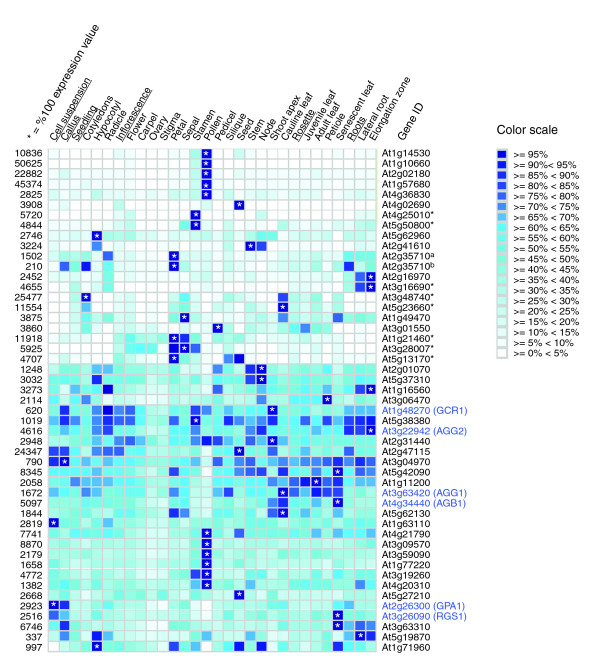
Expression patterns of *Arabidopsis *genes encoding 7TMpR candidates and G-protein subunits among tissues. The figure was modified from an output of the Meta-Analyzer of Genevestigator (last updated in November 2005), which illustrates expression levels of each gene in different organs. Relative expression levels of a gene in different organs/tissues are given as heat maps in blue-scale coding that reflects absolute signal values, where darker colors represent stronger expression. All gene-level profiles are normalized for coloring such that, for each gene, the highest signal intensity obtains a value 100% (shown in the darkest blue and marked with an asterisk) and absence of signal obtains a value 0% (shown in white). All GeneChip data was processed using Affymetrix MAS5.0. Special precaution is required for gene expression in certain cell types (for example, pollen), since difference in normalization may achieve different results. Probe-sets of five 7TMpR candidates (At1g15620. At1g75000, At4g21570, At4g36850, and At5g23990) were not present in the 22K chip, and, therefore, their tissue-specific expression could not be assessed. For At2g35710, two probe-sets (265797_at^a ^and 265841_at^b^) were designed on the chip. Gene names for those belonging to the MtN3 family are shown in boldface and marked with an asterisk. Genes encoding G-protein subunits (*AGB1*, *GPA1*, *AGG1*, and *AGG2*) as well as two reported 7TMpRs (*RGS1 *and *GCR1*) are labeled accordingly in boldface.

We utilized the Meta-Analyzer server of the Genevestigator web site to study spatial expression patterns of *Arabidopsis *genes encoding the 7TMpR candidates and G-protein subunits. Note that the expression of MLO genes were not included in this analysis since we reported them recently [50]. As is shown in Figure 2, expression patterns of analyzed 7TMpR candidates can be divided into two major groups; about half of them show distinct tissue specificity, whereas the other half either exhibit less distinct expression patterns or display ubiquitous expression. All genes encoding G-protein subunits fall into the latter major group. Ubiquitous expression of genes encoding G-protein subunits allows overlap with genes in both groups, and makes, in principle, co-functioning of G-proteins with these 7TMpR candidates spatially and temporally possible. All eight genes encoding the MtN3 family proteins appear to have distinct tissue specific expression. Among them, At3g48740 and At4g25010 have the highest sequence similarities to At5g23660 and At5g50800, respectively. Both pairs of genes share similar or overlapping expression patterns, suggesting relatedness/similarity of their functions. Confirming the actual functions of the 7TMpR candidates as GPCRs requires further extensive testing. A possible involvement of these candidate proteins in 'G protein-independent' signaling mechanisms also needs to be explored.

## Conclusion

We show that the profile HMM protein classification method, currently one of the most used, is overly specific (conservative) when applied to extremely diverged 7TMpR proteins. Our premise is that there are more 7TMpRs yet to be identified in the *A. thaliana *and other genomes divergent to humans. The limitations were that the lack of available samples limits the effectiveness of profile HMM methods, and while alignment-free methods are more sensitive, they have high rates for false positives. The candidate 7TMpR proteins provided in this study, for example, can be included to expand the training set and re-iteration using refined training sets can be done to reduce false positive rates. However, this is possible only after these new candidates are confirmed as true positives experimentally.

The strategy we described here overcomes the 'chicken-or-egg' problem; predictions by multiple protein classification methods and the number of predicted transmembrane regions were used to identify a more likely reduced set of 7TMR candidates. By setting up various methods as hierarchical multiple filters, one can prioritize target protein sets for further experimental confirmation of their functions.

## Materials and methods

### *Arabidopsis *protein data

We downloaded 28,952 protein sequences from TIGR (*Arabidopsis thaliana *database release 5, dated 10 June 2004) [51]. Among the 28,952 proteins, 2,760 are derived from alternative splicing.

### Training data preparation for protein classification

Positive training samples (known 7TMR sequences) were obtained from GPCRDB (Information System for G Protein-Coupled Receptors, Release 9.0, last updated on 28 June 28 2005) [6,7]. In the GPCRDB, 2,030 7TMRs (originally collected from the Swiss-Prot protein database) were grouped into six major classes (classes A to E plus the Frizzled/Smoothened family) and six putative families (ocular albinism proteins, insect odorant receptors, plant MLO receptors, nematode chemoreceptors, vomeronasal receptors, and taste receptors). Five hundred 7TMR sequences were randomly sampled and used as the positive samples. Note that 'putative/unclassified' (orphan) 7TMRs and bacteriorhodopsins were not included in this dataset. These 500 7TMRs included six of the15 known *Arabidopsis *MLO proteins. Among the 22 currently known *Arabidopsis *7TMpRs, in addition to the nine MLO proteins, GCR1 as well as six recently identified *Arabidopsis *7TMpRs (AtRGS1 and HHP1-5; GPCRDB does not list these proteins) were not included in the random 500 7TMR samples. Note that the 15 *Arabidopsis *7TMpRs not included in the training set can be used to assess the classifier performance as test cases.

For negative samples, 500 non-7TMR sequences longer than 100 amino acids were randomly sampled from the Swiss-Prot section of the UniProt Knowledgebase [52,53]. The average length of the 500 non-7TMR sequences was 401 amino acids (with a maximum length of 2,512 amino acids). Positive and negative samples were combined to create a training dataset. Note that only positive samples were used to train the profile HMM classifier, SAM (see below).

### Protein classification methods used

One alignment-based method (profile HMM) and four types of alignment-free multivariate methods were included in our analysis.

#### Profile hidden Markov models

Profile HMMs are full probabilistic representations of sequence profiles [27]. Sample sequences need to be alignable, and thus only positive samples can be used for training. Two programs in SAM (version 3.4) [54,55] were used: *buildmodel *to build profile HMMs with the nine-component Dirichlet mixture priors [56], and *hmmscore *to calculate scores and e-values. The 'calibration' option (for more accurate e-value calculation) and the fully local scoring option (-sw 2) were used. The e-value threshold was set at 0.01 for choosing 7TMR candidates.

#### Discriminant function analysis

Moriyama and Kim [38] described the three parametric (linear, quadratic, logistic) and nonparametric K-nearest neighbor methods that were shown to perform better than the profile HMM method. Therefore, we included these four alignment-free methods (LDA, QDA, LOG, and KNN) in our analysis. For KNN, *K *was set at 5, 10, 15, or 20, where *K *is the number of neighbors. The four variables used (amino acid index and three periodicity statistics) were described in Kim *et al*. [37]. S-PLUS statistical package (Insightful Corporation, Seattle, WA, USA, version 6.1.2 for Linux) with the MASS module [57] was used for the classifier development.

#### Support vector machines with amino acid composition

SVMs are learning machines that make binary classifications based on a hyperplane separating a remapped instance space [58]. A kernel function can be chosen so that the remapped instances on a multidimensional feature space are linearly separable. The radial basis kernel, *exp*(-γ||*x *- *y*||^2^), was used in this study. The parameter γ was set to 102 based on the median of Euclidean distances between positive examples and the nearest negative example as described in Jaakkola *et al*. [59]. Simple 19 amino acid frequencies (the 20th amino acid frequency can be explained completely by the other 19) of each protein sequence were used as an input vector for SVMs. Programs *svm_learn *and *svm_classify *of the SVM^light ^package version 5.0 [60] were used for training and classification, respectively, by SVM. The default value of the regulatory parameter C (0.5006) was used with *svm-learn*. Our comparative analysis showed that SVM-AA performs better than profile HMMs when they are applied to remote similarity identification, the same problem we deal with in this study (PK Strope and EN Moriyama, submitted).

#### Support vector machines with dipeptide composition

We also included an SVM classifier with dipeptide composition [40,41]. The SVM^light ^package version 5.0 [60] was used for training and classification as before. The regulatory parameter C = 1 and the radial basis kernel function parameter γ = 90 were chosen by the grid analysis using 5-fold cross-validation.

#### Partial least squares with amino acid properties

PLS regression is a projection method that takes into account correlations between independent and dependent variables [61]. We used the pls.pcr package, an R implementation developed by Wehrens and Mevik [62,63], with the SIMPLS method, four latent variables, and cross-validation options. Each amino acid in the protein sequences was first converted to a set of 5 principal component scores developed from 12 physico-chemical properties. The auto/cross covariance (ACC) method developed by Wold *et al*. [64] was then applied to each of the converted sequences. ACC describes the average correlations between two residues a certain lag (amino acids) apart. The lag size of 30 was chosen for optimal classification performance. We found that the performance of PLS-ACC is robust even when only a small number of positive samples (5 or 10) are available for training. In contrast, the performance of profile HMMs suffered extremely when positive sample size was small. The 12 physico-chemical properties used and more details on the use of PLS in protein classification are described elsewhere [43]. The cutoff value of 0.4999 was used for choosing 7TMR candidates in this study, which was determined as the average of the minimum error points [39] obtained from 500 replications of 10-fold cross-validation analysis using the training dataset.

### Transmembrane region prediction

HMMTOP 2.0 [46,47] and TMHMM (originally as in [65] but implemented as S-TMHMM by [66]) were used for predicting transmembrane regions. Figure 1 shows the numbers of TM regions predicted by the two methods for the 500 7TMR sequences used for classifier training. HMMTOP predicted 7TM regions from 433 7TMRs (86.6%), while only 165 7TMRs (33%) were predicted to have 7TM regions by TMHMM. HMMTOP predicted 97% or more of 7TMRs to have 6 to 8 TM regions, and with 5 to 9 TM regions more than 99% of 7TMRs were included. Using TMHMM, in order to include 97% of 7TMRs, the range of predicted TM numbers needs to be between 4 and 10. Therefore, we decided to use HMMTOP in our further analysis. With HMMTOP using the range of five to nine TM regions, we should be able to cover almost all possible 7TM proteins.

### Grouping of the candidate proteins

The candidate proteins were grouped based on the e-values obtained by BLASTP protein similarity search [67,68] against the *Arabidopsis *protein database using the default parameter set (for example, BLOSUM62) at the TAIR web site [45]. The e-value threshold of 10^-20 ^was used to identify protein families similar to the candidate proteins.

### Expression patterns of genes encoding 7TMR candidates and G-protein subunits

Expression patterns of genes encoding 7TMpR candidates and G-protein subunits among tissues was studied by using the Meta-Analyzer server of the Genevestigator web site (last updated in November 2005) [69,70]. All data were generated using the 22K Affymetrix ATH1 *Arabidopsis *Genome array. Gene expression profiles based on microarray data were clustered according to similarity in expression patterns. Hierarchical clustering results were generated by default settings using pairwise Euclidean distances and the average linkage method.

## Additional data files

The following additional data files are available with the online version of this paper. Additional data file 1 is the list of the 394 *Arabidopsis thaliana *7TMpR candidates. Additional data file 2 lists the 54 7TMpR candidates identified in this study. These 7TMpR candidates were grouped based on their similarities with known protein families. HTML versions of the candidate lists with TAIR links and other supplementary data are available at [71].

## Supplementary Material

Additional data file 1 The 394 *A. thaliana *7TMpR candidates. Click here for file

Additional data file 2 These 7TMpR candidates were grouped based on their similarities with known protein families. HTML versions of the candidate lists with TAIR links and other supplementary data are available at [72]. Click here for file

## Figures and Tables

**Table 1:** Numbers of 7TMpR candidates identified by various methods from the *A. thaliana *genome

Methods	Number of 7TMpR candidates*
HMMTOP	
7TMs^†^	236 (201)
6-8 TM^†^	633 (545)
5-9 TMs^†^	1,091 (957)
5-10 TMs^†^	1,343 (1,179)
SAM	16 (15)
LDA	3,211 (2,935)
QDA	2,006 (1,820)
LOG	2,626 (2,394)
KNN (*K *= 5)	3,125 (2,839)
KNN (*K *= 10)	3,202 (2,906)
KNN (*K *= 15)	3,298 (3,004)
KNN (*K *= 20)	3,347 (3,043)
SVM-AA	2,263 (2,043)
SVM-di	2,004 (1,807)
PLS-ACC	2,671 (2,466)

*The numbers in parentheses show 7TMpR candidates after removing proteins derived from alternative splicing. ^†^The numbers of TM regions predicted by HMMTOP.

**Table 2:** Summary of the 54 7TMpR candidates identified in this study^1^

Groups*	TAIR locus IDs
Multiple members from gene families	
Nodulin MtN3 family proteins (8/17)	At1g21460, At3g16690, At3g28007, At3g48740, At4g25010, At5g13170, At5g23660, At5g50800
MLO proteins (7/15)	At1g11000 (MLO4), At1g26700 (MLO14), At1g42560 (MLO9), At2g33670 (MLO5), At2g44110 (MLO15), At4g24250 (MLO13), At5g53760 (MLO11)
Expressed protein family 1 (2/6)	At1g77220, At4g21570
GNS1/SUR4 membrane family proteins (3/4)	At1g75000, At3g06470, At4g36830
Perl1-like family protein (2/2)	At1g16560, At5g62130
TOM3 family proteins (3/3)	At1g14530, At2g02180, At4g21790
Expressed protein family 2 (3/5)	At1g10660, At2g47115, At5g62960
Expressed protein family 3 (2/4)	At3g09570, At5g42090
Expressed protein family 4 (2/5)	At1g49470, At5g19870
Expressed protein family 5 (2/5)	At3g63310, At4g02690
Single copy genes (8)	At1g48270 (GCR1), At1g57680, At2g41610, At2g31440, At3g04970, At3g26090 (RGS1), At3g59090, At4g20310
Single member from small gene families (8)	At2g01070, At3g19260, At2g35710, At2g16970, At1g15620, At1g63110, At4g36850, At5g27210
Single member from big gene families (4)	At1g71960, At3g01550, At5g23990, At5g37310

*The number of candidates identified in this study belonging to each group is shown in parentheses (the number of all proteins in each group is given after '/'). More detailed information is given in Additional data file 2.
