# Supplementary material for: Mining the Arabidopsis thaliana genome for highly-divergent seven transmembrane receptors
Source: Genome Biol. 2006 Oct 25;7(10):R96. doi: 10.1186/gb-2006-7-10-r96 (PMC1794564; doi:10.1186/gb-2006-7-10-r96)
Supplement: Additional data file 2 — These 7TMpR candidates were grouped based on their similarities with known protein families. HTML versions of the candidate lists with TAIR links and other supplementary data are available at [72]. [file gb-2006-7-10-r96-S2.pdf]

## Supplementary Table 2. The 54 7TMpR candidates identified by Moriyama, Strope, Opiyo, Chen, and Jones[1]

| Group | ID | Length | Description |
|---|---|---|---|
| **[Multiple members from a big gene family (>5 members)]** | | | |
| **Nodulin MtN3 family** | At1g21460.1 | 247 | 68414.m02683 nodulin MtN3 family protein contains similarity to MTN3 (nodule development protein) GB:Y08726 GI:1619601 from [Medicago truncatula] |
| | At3g16690.1 | 230 | 68416.m02132 nodulin MtN3 family protein contains Pfam PF03083 MtN3/saliva family |
| | At3g28007.1 | 251 | 68416.m03496 nodulin MtN3 family protein contains Pfam PF03083 MtN3/saliva family; similar to LIM7 GI:431154 (induced in meiotic prophase in lily microsporocytes) from [Lilium longiflorum] |
| | At3g48740.1 | 289 | 68416.m05322 nodulin MtN3 family protein similar to MtN3 GI:1619602 (root nodule development) from [Medicago truncatula] |
| | At4g25010.1 | 281 | 68417.m03588 nodulin MtN3 family protein similar to MtN3 GI:1619602 (root nodule development) from [Medicago truncatula] |
| | At5g13170.1 | 292 | "68418.m01508 nodulin MtN3 family protein similar to MtN3 GI:1619602 (root nodule development) from [Medicago truncatula]; identical to cDNA senescence-associated protein (SAG29) mRNA, partial cds GI:4426938" |
| | At5g23660.1 | 285 | 68418.m02774 nodulin MtN3 family protein similar to MtN3 GI:1619602 (root nodule development) from [Medicago truncatula] |
| | At5g50800.1 | 294 | 68418.m06293 nodulin MtN3 family protein similar to MtN3 GI:1619602 (root nodule development) from [Medicago truncatula] |
| **MLO family** | ***At1g11000.1*** | 573 | "68414.m01263 seven transmembrane MLO family protein / MLO-like protein 4 (MLO4) identical to membrane protein Mlo4 [Arabidopsis thaliana] gi\|14091578\|gb\|AAK53797; similar to MLO protein SWISS-PROT:P93766, NCBI_gi:1877221 [Hordeum vulgare][Barley]" |
| | ***At1g26700.1*** | 554 | "68414.m03252 seven transmembrane MLO family protein / MLO-like protein 14 (MLO14) identical to membrane protein Mlo14 [Arabidopsis thaliana] gi\|14091598\|gb\|AAK53807; similar to MLO protein SWISS-PROT:P93766, NCBI_gi:1877221 [Hordeum vulgare][Barley]" |
| | ***At1g42560.1*** | 467 | "68414.m04907 seven transmembrane MLO family protein / MLO-like protein 9 (MLO9) nearly identical to membrane protein Mlo9 [Arabidopsis thaliana] GI:14091588; similar to MLO protein SWISS-PROT:P93766, NCBI_gi:1877221 [Hordeum vulgare][Barley]" |
| | ***At2g33670.1*** | 501 | "68415.m04126 seven transmembrane MLO family protein / MLO-like protein 5 (MLO5) identical to MLO-like protein 5 (AtMlo5) [Arabidopsis thaliana] SWISS-PROT:O22815; similar to MLO protein SWISS-PROT:P93766, NCBI_gi:1877221 [Hordeum vulgare][Barley]" |
| | ***At2g44110.1*** | 496 | "68415.m05485 seven transmembrane MLO family protein / MLO-like protein 15 (MLO15) identical to MLO-like protein 15 (AtMlo15) SP:O80580 from [Arabidopsis thaliana]; similar to MLO protein SWISS-PROT:P93766, NCBI_gi:1877221 [Hordeum vulgare][Barley]" |
| | ***At4g24250.1*** | 478 | "68417.m03480 seven transmembrane MLO family protein / MLO-like protein 13 (MLO13) identical to membrane protein Mlo13 [Arabidopsis thaliana] gi\|14091596\|gb\|AAK53806; similar to MLO protein SWISS-PROT:P93766, NCBI_gi:1877221 [Hordeum vulgare][Barley]" |
| | ***At5g53760.1*** | 573 | "68418.m06680 seven transmembrane MLO family protein / MLO-like protein 11 (MLO11) identical to membrane protein Mlo11 [Arabidopsis thaliana] gi\|14091592\|gb\|AAK53804; similar to MLO protein SWISS-PROT:P93766, NCBI_gi:1877221 [Hordeum vulgare][Barley]" |
| **Expressed protein family 1** | At1g77220.1 | 484 | 68414.m08994 expressed protein contains Pfam profile PF03619: Domain of unknown function |
| | At4g21570.1 | 294 | 68417.m03120 expressed protein contains Pfam profile PF03619: Domain of unknown function |
| **[Multiple members from a small gene family]** | | | |
| **TOM3 family** | At1g14530.1 | 293 | "68414.m01723 tobamovirus multiplication protein 3, putative / TOM3, putative (THH1) identical to THH1 (GI:15706301) [Arabidopsis thaliana]; supporting cDNA gi\|15706300\|dbj\|AB057678.1\|" |
| | At2g02180.1 | 303 | 68415.m00154 tobamovirus multiplication protein 3 (TOM3) identical to tobamovirus multiplication protein (TOM3) GI:15425641 from [Arabidopsis thaliana] |
| | At4g21790.1 | 291 | 68417.m03152 transmembrane protein-related (TOM1) contains some similarity to transmembrane protein TOM3 GI:15425641 from [Arabidopsis thaliana]; identical to cDNA TOM1 GI:9967414 |
| **GNS1/SUR4 membrane family** | At1g75000.1 | 281 | 68414.m08707 GNS1/SUR4 membrane family protein contains Pfam profile PF01151: GNS1/SUR4 family |
| | At3g06470.1 | 278 | 68416.m00749 GNS1/SUR4 membrane family protein similar to SP\|P39540 Elongation of fatty acids protein 1 {Saccharomyces cerevisiae}; contains Pfam profile PF01151: GNS1/SUR4 family |
| | At4g36830.1 | 289 | 68417.m05223 GNS1/SUR4 membrane family protein weak similarity to long chain polyunsaturated fatty acid elongation enzyme [Isochrysis galbana] GI:17226123; contains Pfam profile PF01151: GNS1/SUR4 family |

| | | | |
|---|---|---|---|
| **Expressed protein family 2** | At1g10660.1 | 320 | 68414.m01208 expressed protein |
| | At2g47115.1 | 300 | 68415.m05884 expressed protein |
| | At5g62960.1 | 347 | 68418.m07899 expressed protein |
| **Perl1-like family** | At1g16560.1 | 342 | 68414.m01983 Per1-like family protein contains Pfam profile PF04080: Per1-like |
| | At5g62130.1 | 343 | 68418.m07798 Per1-like protein-related |
| **Expressed protein family 3** | At3g09570.1 | 439 | 68416.m01137 expressed protein |
| | At5g42090.1 | 439 | 68418.m05124 expressed protein |
| **Expressed protein family 4** | At1g49470.1 | 302 | 68414.m05544 expressed protein contains Pfam profile PF04819: Family of unknown function (DUF716) (Plant viral-response family) |
| | At5g19870.1 | 276 | 68418.m02363 expressed protein contains Pfam profile PF04819: Family of unknown function (DUF716) (Plant viral-response family) |
| **Expressed protein family 5** | At3g63310.1 | 239 | 68416.m07121 expressed protein low similarity to N-methyl-D-aspartate receptor-associated protein [Drosophila melanogaster] GI:567104; contains Pfam profile PF01027: Uncharacterized protein family UPF0005 |
| | At4g02690.1 | 248 | "68417.m00364 hypothetical protein low similarity to N-methyl-D-aspartate receptor-associated protein [Drosophila melanogaster] GI:567104, NMDA receptor glutamate-binding subunit [Rattus sp.] GI:8248741; contains Pfam profile PF01027: Uncharacterized prote" |
| **[Single copy genes]** | | | |
| **GCR1** | ***At1g48270.1*** | 326 | 68414.m05392 G protein coupled receptor-related identical to putative G protein coupled receptor GI:2104224 from [Arabidopsis thaliana] |
| | At1g57680.1 | 362 | 68414.m06545 expressed protein |
| | At2g41610.1 | 310 | 68415.m05142 expressed protein |
| | At2g31440.1 | 250 | 68415.m03841 expressed protein identical to cDNA endonuclease III homologue (nth1 gene) GI:11181951 |
| | At3g04970.1 | 397 | 68416.m00540 zinc finger (DHHC type) family protein similar to Golgi-specific DHHC zinc figer protein [Mus musculus] GI:21728103; contains Pfam profile PF01529: DHHC zinc finger domain |
| **RGS1** | ***At3g26090.1*** | 459 | 68416.m03249 expressed protein |
| | At3g59090.1 | 367 | 68416.m06587 expressed protein |
| | At4g20310.1 | 513 | 68417.m02966 peptidase M50 family protein / sterol-regulatory element binding protein (SREBP) site 2 protease family protein weak similarity to SP\|O54862 Membrane-bound transcription factor site 2 protease (EC 3.4.24.-) (Sterol-regulatory element-binding |
| **[Single member from a small gene family]** | | | |
| | At2g01070.1 | 496 | 68415.m00013 expressed protein similar to membrane protein PTM1 precursor isolog GB:AAB65479 |
| | At3g19260.1 | 296 | 68416.m02443 longevity-assurance (LAG1) family protein similar to Alternaria stem canker resistance protein (ASC1) [Lycopersicon esculentum] GI:7688742; contains Pfam profile PF03798: Longevity-assurance protein (LAG1) |
| | At2g35710.1 | 497 | 68415.m04380 glycogenin glucosyltransferase (glycogenin)-related low similarity to glycogenin-2 from Homo sapiens [SP\|O15488] |
| | At2g16970.1 | 414 | 68415.m01955 expressed protein ; expression supported by MPSS |
| | At1g15620.1 | 343 | 68414.m01877 expressed protein ; expression supported by MPSS |
| | At1g63110.2 | 397 | 68414.m07132 cell division cycle protein-related contains 9 transmembrane domains; similar to PIG-U (GI:27372215) [Rattus norvegicus]; similar to Cell division cycle protein 91-like 1 (CDC91-like 1 protein) (PIG-U) (Swiss-Prot:Q9H490) [Homo sapiens] |
| | At4g36850.1 | 374 | 68417.m05225 PQ-loop repeat family protein / transmembrane family protein similar to SP\|Q10482 Seven transmembrane protein 1 {Schizosaccharomyces pombe}; contains Pfam profile PF04193: PQ loop repeat |
| | At5g27210.1 | 297 | 68418.m03246 expressed protein weak similarity to seven transmembrane domain orphan receptor [Mus musculus] GI:4321619 |
| **[Single member from a large gene family]** | | | |
| | At1g71960.1 | 662 | 68414.m08318 ABC transporter family protein similar to breast cancer resistance protein GB:AAC97367 from [Homo sapiens] |
| | At3g01550.1 | 383 | "68416.m00085 triose phosphate/phosphate translocator, putative similar to SWISS-PROT:P52178 triose phosphate/phosphate translocator [Cauliflower]{Brassica oleracea}" |
| | At5g23990.1 | 657 | "68418.m02819 ferric-chelate reductase, putative similar to ferric-chelate reductase (FRO1) [Pisum sativum] GI:15341529; contains Pfam profile PF01794: Ferric reductase like transmembrane component" |
| | At5g37310.1 | 564 | "68418.m04481 endomembrane protein 70, putative multispanning membrane protein, Homo sapiens, EMBL:HSU94831" |

**[1]7TMpR candidates were grouped based on their BLASTP E-values of $10^{-20}$ or better. Sequence IDs of nine previously reported 7TMpRs including 7 MLOs, GCR1, and RGS1 are shown in boldface and italic.**
